# Response to: “Monosodium urate crystal deposition associated with the progress of radiographic grade at the sacroiliac joint in axial SpA: a dual-energy CT study”

**DOI:** 10.1186/s13075-018-1524-0

**Published:** 2018-02-12

**Authors:** Andrea S. Klauser, Johann Gruber

**Affiliations:** 1 Department of Radiology, Medical University Innsbruck, Section Rheumatology and Sports Imaging, Anichstrasse 35, A-6020 Innsbruck, Austria; 2 Department of Rheumatology, Medical University Innsbruck, Anichstrasse 35, A-6020 Innsbruck, Austria; 3 Department of Internal Medicine II, Medical University Innsbruck, Anichstrasse 35, 6020 Innsbruck, Austria

**Keywords:** Dual-energy computed tomography, Sacroiliitis, Monosodium urate, Axial spa

We read with great interest the article by Zhu et al. [1] entitled “**Monosodium urate crystal deposition associated with the progress of radiographic grade at the sacroiliac joint in axial SpA: a dual-energy CT study”** which was published in *Arthritis Research & Therapy* in May 2017. We congratulate the authors on attempting to verify gouty deposits at the sacroiliac joint in axial SpA patients using dual-energy computed tomography (DECT), a relatively new imaging method to detect gouty deposits.

Deposition of monosodium urate (MSU) in the spine is a rare manifestation of gout, and only case and series reports exist in the literature [2].

Axial SpA patients without gout and with no hyperuricemia were included in this study; however, a case–control group would be of interest to compare results with gout patients and with nonaxial SpA patients in order to elucidate the prevalence of spinal gout involvement, which is actually unknown [2].

In addition, matters of concern arise when considering the presented figures.

DECT artifacts according to ACR/EULAR guidelines have to be differentiated from gouty deposits when submillimeter deposits, skin deposits, deposits obscured by motion, beam hardening, and vascular artifact are present [3, 4].

Submillimeter artifacts may be single or may form part of a diffuse pattern of the scatter. They are thought to occur as a result of and as a form of noise [4].

Furthermore, it has been shown recently using DECT that MSU crystal deposition is generally present within the joint, on the bone surface, and within bone erosion, but is not observed within bone in the absence of a cortical break [5]. Interestingly, the green DECT pixels presented in the figures (rated as MSU deposits) are mainly depicted inside the sacrum and the iliac bone, and not in the sacroiliac joint nor pronounced on the bone surface. This is contrary to the “bone cortex concept” where MSU crystals deposit outside bone and contribute to bone erosion through an “outside-in” mechanism [5].

## Abbreviations

DECT: dual-energy computed tomography
MSU: monosodium urate
